# Enzymatic Characterization of *SpPAL* Genes in *S. polyrhiza* and Overexpression of the *SpPAL3*

**DOI:** 10.3390/plants13182607

**Published:** 2024-09-18

**Authors:** Xiaoxue Li, Yinxing Zhang, Chunfeng Zhu, Pufan Zheng, Cunkun Chen, Na Zhang, Haipeng Ji, Chenghu Dong, Jinze Yu, Jie Ren, Yerong Zhu, Yong Wang

**Affiliations:** 1 Institute of Agricultural Products Preservation and Processing Technology, National Engineering Technology Research Center for Preservation of Agriculture Product, Tianjin Academy of Agricultural Sciences, Tianjin 300384, China; m15822139395@163.com (X.L.); zhengpufan@foxmail.com (P.Z.); cck0318@126.com (C.C.); wuaidehua@163.com (N.Z.); crazysum41@163.com (H.J.); dongchenghu@sina.com (C.D.); yujinze74@126.com (J.Y.); 2 School of Life Science, Tianjin University, Tianjin 300072, China; yinxing.zhang@tju.edu.cn (Y.Z.); zhuchunfeng0613@tju.edu.cn (C.Z.); 3 College of Food Science and Biological Engineering, Tianjin Agricultural University, Tianjin 300392, China; renjie211@163.com; 4 College of Life Science, Nankai University, Tianjin 300071, China

**Keywords:** *Spirodela polyrhiza*, phenylalanine ammonia-lyase, cloning, characterization, overexpression

## Abstract

Phenylalanine ammonia-lyase (PAL, EC 4.3.1.5) catalyzes the deamination of phenylalanine, which is the initial step in the biosynthesis of phenylpropanoids. It serves as a crucial enzyme that facilitates the transfer of carbon from primary to secondary metabolism in plants. Duckweed is regarded as a promising chassis plant in synthetic biology research and application, due to its being rich in secondary metabolites and other advantages. The genes encoding PAL in *Spirodela polyrhiza* (L.) Schleid, the giant duckweed, were investigated in this study. Three *SpPAL* genes (*SpPAL1–SpPAL3*) were identified and cloned. All of them were successfully expressed in *E. coli*, and their recombinant proteins all showed PAL activity. In addition, SpPAL1 and SpPAL2 proteins could also utilize tyrosine as substrate, although the activity was low. A qRT-PCR analysis demonstrated that the expression of *SpPAL3* was most pronounced in young fronds. It was found that the expression of *SpPAL1* and *SpPAL3* was significantly induced by MeJA treatment. Overexpression of *SpPAL3* in *Lemna turionifera* inhibited the growth of fronds and adventitious roots in the transgenic plants, indicating the importance of *SpPAL3* in duckweed besides its involvement in the secondary metabolism.

## 1. Introduction

Synthetic biology represents a significant advancement of recombinant DNA technology, offering new capabilities for the creating, assembling, modifying, and controlling of different genes or biological systems [1]. Plant synthetic biology, unlike synthetic biology which primarily involves microorganisms as hosts, offers superior systems for synthesizing bioactive properties derived from plants [2,3]. Research in plant synthetic biology is booming [4,5].

Species of the *Lemnaceae* family, known as duckweeds, are the smallest and fastest-growing aquatic flowering plants investigated to date [6]. The plant exhibits an extended annual production cycle characterized by a rapid increase in biomass generation, which is higher than the growth rates of many other plant species. In regions with warm climates, the production output can reach up to 12 g/m^2^/day of dry weight [7]. Under specific circumstances, the asexual reproduction of duckweed leads to genetic stability, thereby minimizing the occurrence of genetic drift [8]. In many regions of the world, duckweed is commonly used as a sustainable feedstock for biofuel production and as fodder for ducks because it is rich in starch and protein [7,9]. In addition to having high starch and protein contents, duckweed plants are also rich in flavonoids, particularly the duckweed strain *Spirodela polyrhiza* [10]. Luteolin-8-C-glucoside, luteolin-7-O-glucoside, and apigenin-7-O-glucoside are all flavonoids derived from the phenylpropanoid pathway in *S. polyrrhiza* [11]. In recent years, duckweed plants have been developed as chassis plants in synthetic biology due to their simple structure, fast growth, and richness in secondary metabolites [12]. It was reported that duckweed as a synthetic biology chassis co-expressed *CFP-AtERI1*, *MmDGAT,* and a sesame oleosin variant, achieving an increase in TAG by 7- to 45-fold compared to the WT [13]. However, the use of duckweed as a chassis in synthetic biology for the production of natural plant products has been limited. Therefore, it is necessary to identify and clone genes involved in the pathway of secondary metabolisms in duckweed plants, as well as to elucidate the related metabolic network.

Phenylpropanoid biosynthesis is critically important for plant development, growth, disease resistance, mechanical support, and environmental adaptation [14]. It is responsible for the production of many aromatic metabolites in plants. At present, over 8000 different aromatic compounds have been discovered [15]. Phenylalanine ammonia-lyase (PAL, EC 4.3.15) is responsible for the initial step in the biosynthesis of phenylpropanoids and functions as the key enzyme in this metabolic pathway, leading the conversion of L-phenylalanine from the main carbon reservoir towards the synthesis of *trans*-cinnamic acid (*t*-CA). Subsequently, the generated *t*-CA undergoes further conversions to produce various phenolic compounds [14]. Currently, identifying and cloning of *PAL*-encoding genes have been reported extensively, and it was demonstrated that in the majority of plant species, PAL is produced by a gene family. To date, however, *PAL* genes involved in phenylpropanoid biosynthesis in *S. polyrrhiza* have not been cloned and experimentally investigated.

In this study, genome and transcriptome data of *S. polyrrhiza* were used to identify genes encoding PAL. The identified genes were cloned and expressed in *E. coli* to characterize their biological functions. The expression of these *PAL*-encoding genes in different tissues and the effect of methyl jasmonate (MeJA) on *S. polyrrhiza* were investigated. *SpPAL3* overexpression in *Lemna turionifera* was obtained to verify the role of *SpPAL3* in duckweed.

## 2. Results

### 2.1. Cloning of SpPALs

Three *SpPAL* genes were annotated in the *S. polyrhiza* whole genome [16] and named *SpPAL1*, *SpPAL2*, and *SpPAL3*, with full-length cDNA sequences of 2172 bp, 2277 bp, and 2196 bp, respectively. Specific primers (Supplementary Table S1) were designed and used to clone the three full-length cDNA by RT-PCR. After sequencing, sequences were deposited in GenBank with the accession numbers MW269620, MW269621, and MW269622, respectively, and alignments were performed, which showed that all have a high identity with other reported *PALs* (Supplementary Figure S1).

### 2.2. Bioinformatics and Phylogenetic Analyses of SpPALs

The deduced protein sequences of SpPALs were compared with homologous proteins deposited in GenBank through multiple sequence alignment (Supplementary Figure S1), showing that SpPALs exhibit high similarity with other PALs. *SpPAL1* is more similar to *SpPAL3* (86%) than to *SpPAL2* (62%). The three highly homologous PALs are found in *Lycoris radiata* (AWW24969.1, PAL), *Zea mays* (ONM07141.1, PAL1), and *Picrorhiza kurrooa* (AGA84059.1, PAL). The SpPAL proteins varied in length, from 723 to 758 amino acids, with predicted molecular weights ranging between 78.032 kDa and 83.592 kDa, as outlined in Table 1. The isoelectric points of the three SpPAL proteins were six. Analysis of subcellular localization, transmembrane helices, and signal peptide prediction were performed using CELLO, TMHMM Server v. 2.0, and SignalP-5.0 Server. The results indicate that all three SpPALs are localized in the cytosol and do not possess transmembrane helices or signal peptides (Table 1).

In order to investigate the conserved segments of the SpPAL proteins, an analysis of the motifs present in three SpPAL proteins was conducted using the MEME online tool. A total of twenty conserved motifs were detected, as detailed in Supplementary Table S2. The results further reveal that all SpPALs exhibit the conserved PAL protein finger motif (GTITASGDLVPLSYIA), a distinctive hallmark of PAL/HAL proteins, as illustrated in Supplementary Figure S1. Another conserved residue “FL” (highlighted in the red box) contributing to the substrate selectivity switch was found in SpPALs, suggesting that SpPALs are typical MIO-dependent enzymes (Supplementary Figure S1). These two locations are believed to have a significant impact on the functionality of this particular protein [17].

By utilizing the SOPMA online program, the secondary structure prediction of SpPAL1, SpPAL2, and SpPAL3 revealed that α-helices (54.5%, 53.69%, and 55.13%) were the predominant structural elements, and random coils (30.43%, 32.45%, and 30.78%) were distributed throughout the entire proteins. To better characterize the SpPAL1, SpPAL2, and SpPAL3 proteins, comparative modeling of tertiary structure was performed using SWISS-MODEL (Supplementary Figure S2), which reveals that the tertiary structures also exhibit a ‘sea horse’ shape. At the same time, by using the known *Petroselinum crispum* (1w27.1) as a template to build tertiary structures, the results show that these three SpPAL proteins maybe are all homotetramers.

**Figure 1:**
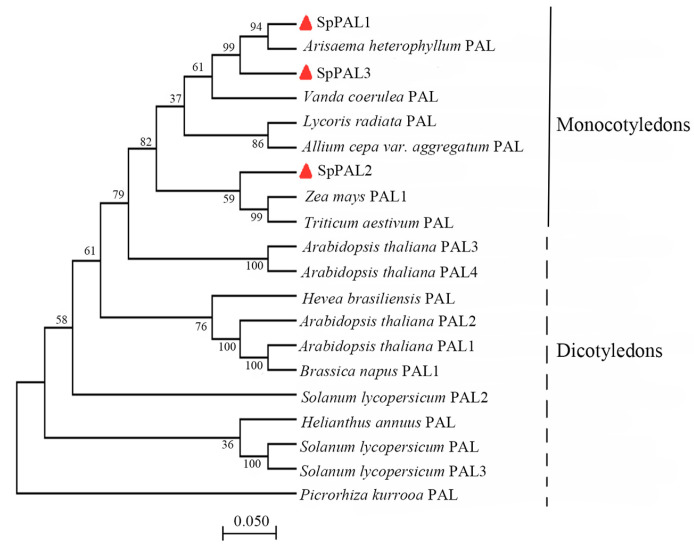
Phylogenetic analyses of SpPALs in duckweeds. Phylogenetic analysis of SpPALs with other PAL proteins. The numerical values displayed on the internal branches represent the bootstrap values derived from 1000 replicates. The bar represents a genetic divergence of 0.050. Plant species and GenBank accession numbers of their PAL proteins used for phylogenetic analysis are as follows: *Arabidopsis thaliana* PAL1 (P35510.3), *Arabidopsis thaliana* PAL2 (NP_190894.1), *Arabidopsis thaliana* PAL3 (NP_001190223.1), *Arabidopsis thaliana* PAL4 (NP_187645.1), *Brassica napus* PAL1 (NP_001303114.1), *Helianthus annuus* PAL (CAA73065.1), *Hevea brasiliensis* PAL (AQD20651.1), *Lycoris radiata* PAL (AWW24969.1), *Picrorhiza kurrooa* PAL (AGA84059.1), *Solanum lycopersicum* PAL (AAA34179.2), *Solanum lycopersicum* PAL2 (NP_001307530.1), *Solanum lycopersicum* PAL3 (NP_001307538.1), *Vanda coerulea* PAL (AYI63776.1), *Zea mays* PAL1 (ONM07141.1), *Arisaema heterophyllum* PAL (QDX15853.1), *Allium cepa var. aggregatum* PAL (AYV89932.1), and Triticum aestivum PAL (QNR01086.1).

To investigate the evolutionary relationship between SpPALs and PALs from other plants, we compiled a set of PAL amino acid sequences from various plant species. This set included 11 dicots and 9 monocots, with *Picrorhiza kurrooa* serving as the outgroup. Subsequently, we generated a phylogenetic tree using the neighbor-joining approach in MEGA 7.0. The structure of the phylogenetic tree typically aligns well with the conventional taxonomic classification. Phylogenetic analysis divided the PAL family into two groups, monocotyledons and dicotyledons, and the SpPAL proteins were clustered in the monocotyledons group (Figure 1). Interestingly, SpPAL1 and SpPAL3 have a high degree of similarity with PAL from *Arisaema heterophyllum*, while SpPAL2 has a high degree of similarity with PAL from *Zea mays* and *Triticum aestivum*, showing that the SpPAL2 may have a different evolution pathway from the two other SpPALs.

### 2.3. Expression and Purification of Recombinant SpPALs

**Figure 2:**
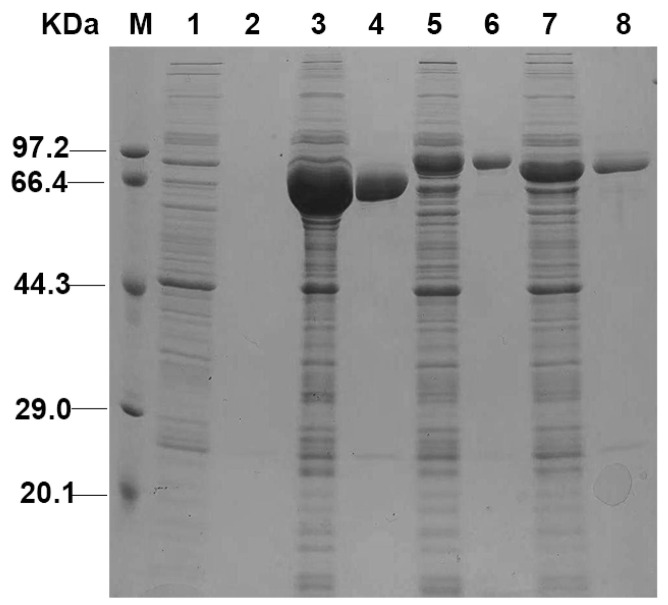
SDS-PAGE analysis of recombinant SpPALs expression and purification. M: Protein molecular mass marker; 1: pET-28a (+) in *E. coli* Rosetta (DE3); 2: purified pET-28a (+); 3, 5, and 7: pET-28a (+) -SpPAL1, 2, and 3 in *E. coli* Rosetta (DE3), respectively; 4, 6, and 8: purified recombinant SpPAL1, 2, and 3, respectively.

To express SpPAL proteins in *E. coli* and reveal their catalytic properties, three recombinant plasmids were constructed using the pET28a (+) vector. A (His) 6-tag was fused to the N-terminus to facilitate the purification of the protein. The recombinant (His) 6-tagged SpPAL proteins were purified on a Ni–IDA-chelating column and eluted using a 250 mM imidazole buffer. SpPALs were successfully expressed in *E. coli*. According to SDS-PAGE analysis, the molecular weights of recombinant SpPALs were approximately 78 kDa, 83 kDa, and 79 kDa, respectively (Figure 2), which is in good agreement with the sizes predicted by the bioinformatics method.

### 2.4. Functional Characterization of Recombinant SpPALs

**Figure 3:**
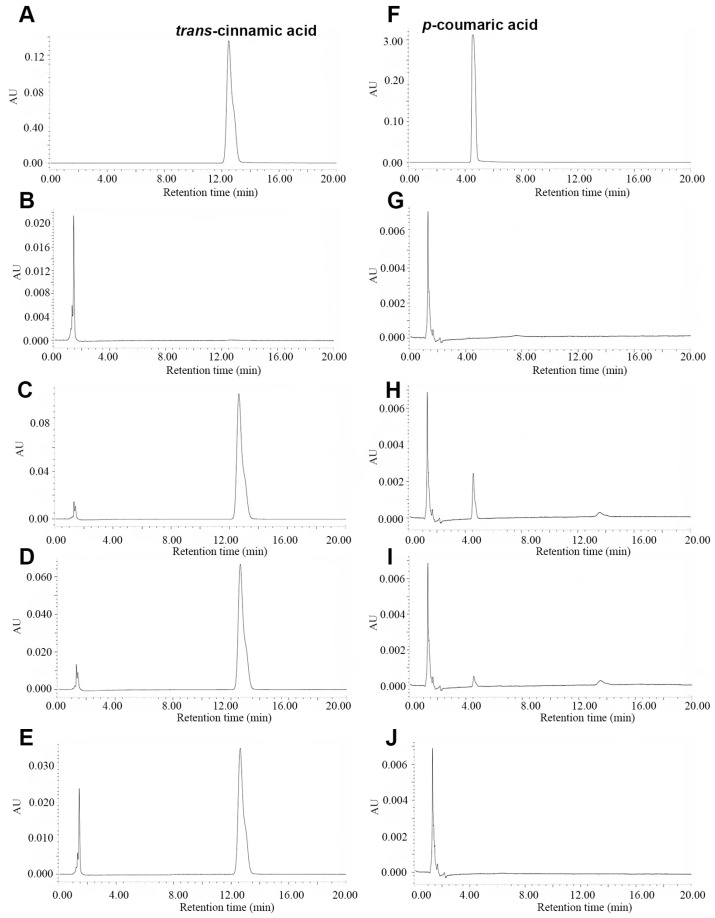
HPLC chromatograms showing SpPALs-catalyzed reaction. (**A**). Standard *p*-coumaric acid; (**B**). pET-28a (+) enzymatic reaction added with L-Phe; (**C**–**E**). Enzymatic reactions catalyzed by purified SpPAL1, 2, and 3, respectively, and added with L-Phe; (**F**). Standard *p*-coumaric acid; (**G**). pET-28a (+) enzymatic reaction added with L-Tyr; (**G**–**J**). The same as (**C**–**E**), added with L-Tyr instead of L-Phe.

PALs are reported to catalyze the conversion of L-Phe to *trans*-cinnamic acid and, in some cases, of L-Tyr to *p*-coumaric acid. To validate the function of SpPALs, the protein was incubated with L-Phe or L-Tyr in the reaction solution. After the reaction was terminated, the product (*trans*-cinnamic acid or *p*-coumaric acid) was detected using HPLC (Figure 3). The results show that three recombinant SpPALs can catalyze the convertion of L-Phe to *trans*-cinnamic acid. In addition, SpPAL1 and SpPAL2 can catalyze the conversion of L-Tyr to *p*-coumaric acid, although the yield of *p*-coumaric acid was very low. These results demonstrate that SpPAL3 can use L-Phe as a substrate.

**Figure 4:**
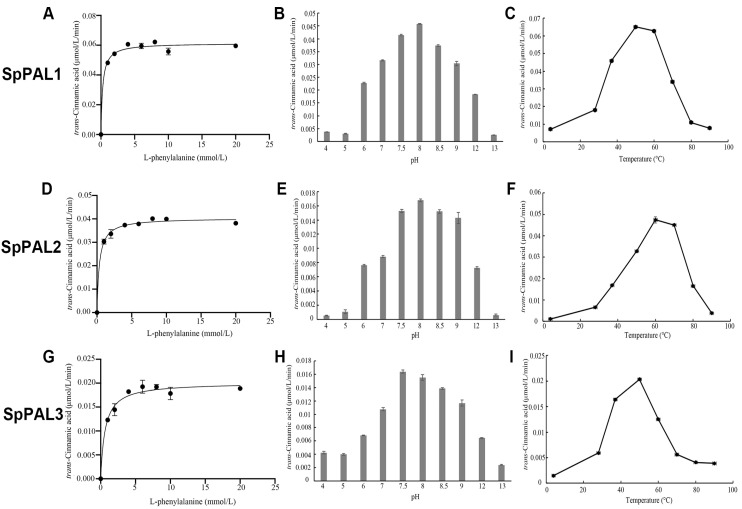
Biochemical characterization of the recombinant SpPALs and determination of kinetic properties of SpPALs using L-Phe as a substrate. (**A**,**D**,**G**): Kinetic parameters of SpPAL1, 2, and 3, respectively; (**B**,**E**,**H**): Optimal pH analysis of recombinant SpPAL1, 2, and 3, respectively; (**C**,**F**,**I**): Optimal temperature analysis of recombinant SpPAL1, 2, and 3, respectively.

To compare the biochemical properties of the three recombinant SpPALs, we measured the Michaelis–Menten kinetics (Figure 4) and other enzymatic parameters, including the kinetic parameters, pH, and temperature optima (Table 2).

Using L-Phe as a substrate, it was determined that the optimal pH and temperature for the activity of SpPAL1 were 8.0 and 50 °C, respectively (Figure 4B,C); for the activity of SpPAL2, they were 8.0 and 60 °C, respectively (Figure 4E,F); and for the activity of SpPAL3, they were 7.5 and 50 °C, respectively (Figure 4H,I). The kinetic parameter results demonstrate that SpPAL1 is the most effective among these three SpPALs (Figure 4A,D,G). The Km value of SpPAL3 for L-Phe was the highest, at 632 μM; while that of SpPAL2 was higher, at 345 μM, compared to SpPAL1, at 252 μM. Higher Kcat values were observed for SpPAL1 with L-Phe as a substrate (9.83 s^−1^) compared to those of SpPAL2 (7 s^−1^) and SpPAL3 (3.35 s^−1^). This resulted in the highest Kcat/Km value in SpPAL1 towards L-Phe (39,008 s^−1^ M^−1^), highlighting the higher catalytic efficiency of SpPAL1 over the other two SpPALs.

### 2.5. Transcription Profile of SpPALs in Different Tissues and under MeJA Treatment

**Figure 5:**
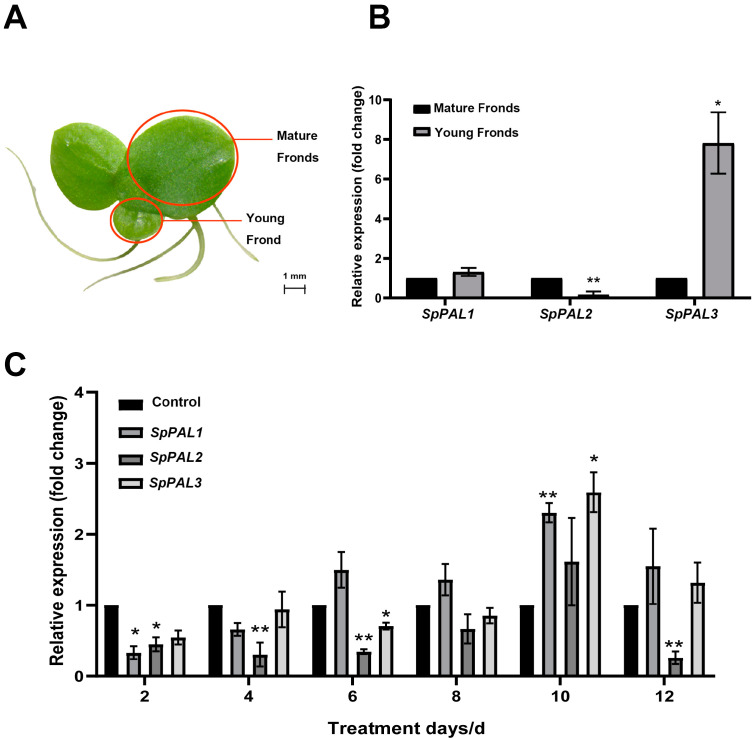
Expression analysis of *SpPALs* in different tissues and under MeJA treatment in duckweed. (**A**). A brief sketch of the various tissues of duckweed is provided; (**B**). *SpPALs* transcript levels in various tissues were determined through qRT-PCR analysis; (**C**). Expression patterns of *SpPALs* under 100 μM MeJA on different days. Control: *INO1* gene. Statistical significance was assessed through one-way ANOVA and Student’s *t*-test (* *p* < 0.05, ** *p* < 0.01).

In order to analyze the levels of expression of the *SpPAL* genes across various tissues, the inositol-3-phosphate synthase (*INO1*) gene was used as an endogenous reference gene. A qRT-PCR was conducted using primers specific to each gene (please refer to Supplementary Table S1). The expression levels of the *SpPAL* genes were distinctly different in mature fronds and young fronds (Figure 5A,B). *SpPAL1* exhibited a slightly higher transcriptional level in young fronds. *SpPAL2* had a lower transcription level in young fronds. *SpPAL3* had the highest expression level in the young fronds, being up-regulated 7.81 times compared to mature fronds.

Furthermore, due to the significance of *PAL* in the plant stress response, MeJA elicitation experiments were conducted to investigate the alteration in expression levels (Figure 5C). As shown in Figure 5C, with the extension of the MeJA treatment time, the *SpPALs* exhibited a pattern of initially decreasing, then increasing, and finally decreasing again. At the same time, on the tenth day of treatment, the expression levels of *SpPAL1*, *SpPAL2*, and *SpPAL3* reached their peak compared to the control group, being 2.3, 1.6, and 2.6 times higher, respectively. After MeJA treatment, the expression level of *SpPAL2* increased on the tenth day; however, at other time points, it was lower than that of the control group. Based on the low expression level of *SpPAL2* in the *S. polyrhiza* mentioned above, it is suggested that *SpPAL2* may not be the key gene for resisting external stimuli in *S. polyrhiza*.

### 2.6. Overexpression of SpPAL3 in Lemna turionifera

**Figure 6:**
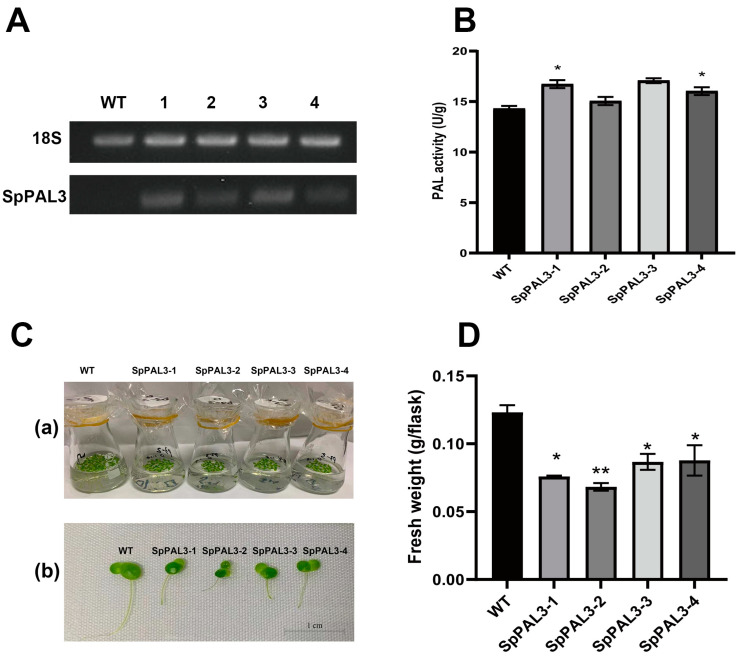
Overexpression of *SpPAL3* in *L. turionifera* under normal conditions. (**A**). Expression of *SpPAL3* mRNA was tested by RT-PCR in four transgenic plants and WT plants, 18S:*18S* rRNA, which was used as the internal control; (**B**). PAL activity of four transgenic plants and WT plants; (**C**). phenotype of *SpPAL3*-overexpressed duckweeds and WT plants, (a) phenotype of *SpPAL3*-overexpressed duckweeds and WT plants in medium, (b) phenotype of *SpPAL3*-overexpressed duckweeds and WT plants under a microscope; (**D**). fresh weight of four transgenic plants and WT plants. Statistical significance was assessed through one-way ANOVA and Student’s *t*-test (* *p* < 0.05, ** *p* < 0.01).

In the results mentioned above, it was suggested that SpPAL3 only has PAL activity and may participate in the growth, development, and response to the stress in duckweed. In order to investigate the impact of *SpPAL3* in duckweed, a transgenic approach was employed in *L. turionifera* plants that were engineered to express the *SpPAL3*, as illustrated in Figure 6. RT-PCR was performed to analyze the transcript levels of *SpPAL3* in the transgenic duckweed and the wild-type (WT) duckweed. As the results show, *SpPAL3* was expressed in four transgenic duckweed lines (Figure 6A). The PAL enzyme activity in four transgenic duckweed lines was slightly higher than in the WT line (Figure 6B). In typical circumstances, a clear difference in physical characteristics was evident between genetically modified plants and WT plants (Figure 6C). The fronds of transgenic plants were smaller than those of WT plants, and the adventitious roots were shorter than those of WT plants. Concurrently, the transgenic plants showed a significant decrease in fresh weight compared to the WT plants (Figure 6D). These findings indicate that the increased expression of *SpPAL3* in duckweed may inhibit the growth of fronds and adventitious roots.

**Figure 7:**
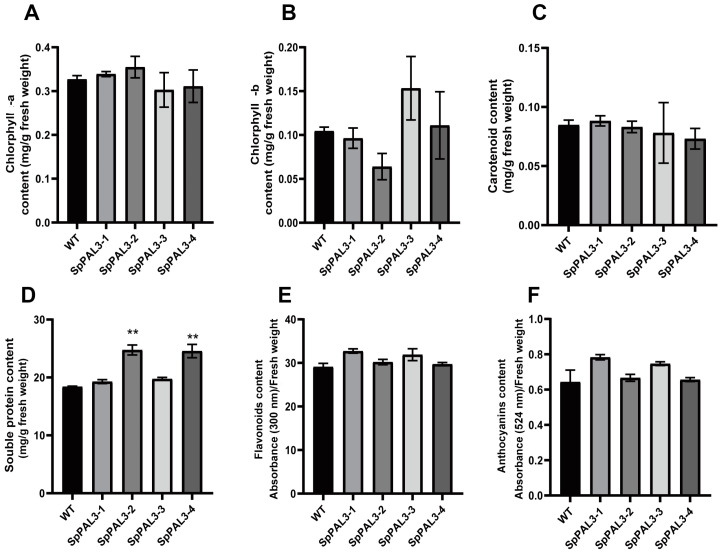
Physiological and biochemical indices of *SpPAL3*-overexpressed and WT duckweeds under normal conditions. (**A**–**C**). Chlorphyll-a, chlorphyll-b, and carotenoid content were measured in four transgenic plants and WT plants, respectively; (**D**). soluble protein content of four transgenic plants and WT plants; (**E**,**F**). flavonoids and anthocyanins content of four transgenic plants and WT plants, respectively. Statistical significance was assessed through one-way ANOVA and Student’s *t*-test (** *p* < 0.01).

To further analyze whether *SpPAL3* has another function in duckweeds, we measured the pigment content, soluble protein content, flavonoid content, and anthocyanin content in *SpPAL3*-overexpressed duckweeds and WT plants (Figure 7). As shown in Figure 7A–C, there was no significant difference in chlorphyll-a, chlorphyll-b, and carotenoid content between WT plants and transgenic plants. The soluble protein content of transgenic duckweed lines 2 and 4 was higher than that of WT plants (Figure 7D). The levels of flavonoids and anthocyanins in the four transgenic duckweed lines did not show any significant variance when compared to those in WT plants, as depicted in Figure 7E,F. Interestingly, it was observed that the concentration of soluble proteins in transgenic lines 2 and 4 exceeded that in lines 1 and 3. And the flavonoids and anthocyanins content of transgenic lines 2 and 4 were lower than that of lines 1 and 3.

## 3. Discussion

The enzyme PAL serves as the initial regulatory step in the phenylpropanoid pathway and plays a crucial role in the physiological processes of plant growth and development [18,19]. It initiates the process of transforming L-Phe into *trans*-cinnamic acid, which is subsequently converted by C4H (cinnamic acid-4-hydroxylase) into *p*-coumaric acid, serving as the precursor for various phenolic compounds [14,20]. Furthermore, it has been documented that PALs derived from various monocotyledonous plants exhibit tyrosine ammonia-lyase (TAL) activity and utilize tyrosine as a substrate, such as *Bambusa oldhamii* [21], *Phyllostachys edulis* [22], and *Zea mays* [23]. Studies on PAL enzyme properties and the characterization of the encoding genes from various plants have been extensively documented in the literature, and this general knowledge has been consistently reaffirmed. However, no detailed studies of PAL/TAL activity in duckweed have been conducted.

In the majority of plant species, PAL is produced by a group of multiple genes. For instance, *Arabidopsis thaliana* (4 PALs) [24], *Marchantia polymorpha* (10 PALs) [25], and tomato (26 PALs) [26] contain several *PAL* gene members. In this study, we characterized three *PAL*-encoding genes in *S. polyrhiza*. Three *PAL*-encoding genes have also been described in another species of duckweed, *Landoltia punctata* [7]. However, it cannot be ruled out that there may be other PAL family members in duckweed plants. The full-length cDNA sequences indicate that *SpPAL1*, *SpPAL2*, and *SpPAL3* encode proteins of 723, 758, and 731 amino acids, respectively, which are similar to those of other reported PALs [27]. PAL is normally believed to be a homotetrameric protein consisting of four identical subunits [28], with a molecular weight of approximately 275–330 kDa in vivo (Table 1) [15]. Building the tertiary structure reveals that all three SpPAL proteins maybe are also homotetramers (Figure S2).

A conserved Lyase aromatic domain was found in most PALs, which helps regulating the growth and development of plants [29]. Sequence analysis and homology modeling reveal that SpPALs share identical characteristics with many other PALs (Figure S1). In plants, PAL proteins belong to the MIO-dependent enzyme family [30] and possess a conserved Ala–Ser–Gly catalytic triad that can be converted into the MIO prosthetic group [14,15]. The Ala–Ser–Gly catalytic triad is also present in all SpPALs (Figure S1), indicating that SpPALs have similar post-transcriptional regulation of enzyme activity. Using the SignalP-5.0 program, no signal peptide was detected for the SpPAL proteins, indicating that SpPALs is probably cytoplasmic localization (Table 1). These results of SpPALs were similar to those of PALs from *Fritillaria unibracteata* [31] and *Peucedanum praeruptorum Dunn* [32].

The various isoforms of PAL may have distinct roles in different branches of the phenylpropanoid pathway [33,34]. In *Arabidopsis thaliana*, the four isoforms exhibit varying degrees of activity: AtPAL1, 2, and 4 demonstrate robust catalytic activity, while AtAPL3 shows limited enzyme activity [35]. In *Lycoris radiata*, it has been reported that LrPAL1 and LrPAL2 are structurally similar but have different biological functions [33]. Within phylogenetic trees, the SpPAL1, SpPAL3, and SpPAL2 sequences are not closely related to each other phylogenetically (Figure 1), indicating that they did not arise from a simple gene duplication event. This may indicate that SpPAL1, SpPAL2, and SpPAL3 have different functions in duckweed.

For the recombinant SpPAL proteins, the characterization of the kinetic and physical parameters was further analyzed; the results are shown in Figure 2, Figure 3 and Figure 4. The recombinant SpPAL proteins extracted were incubated with L-Phe in the reaction buffer according to the method described in M and M, and the product was detected via HPLC (Figure 3A–E). The results confirmed that all three SpPALs can catalyze the forward deamination, which is responsible for the conversion of L-Phe to *trans*-cinnamic acid. They have similar optimal reaction temperatures (50–60 °C) and pH levels (7.5–8.0) ranges showed in Figure 4, which were not significantly different from other plant PALs [35,36]. The Km value of *SpPAL1* was determined to be the lowest (Table 2). Moreover, PAL enzymes derived from various sources, especially fungi and monocots, exhibit enzymatic activity towards L-Tyr, resulting in the synthesis of *p*-coumaric acid. This phenomenon is commonly referred to as TAL activity [14,28]. Among all four PALs from *Bambusa oldhamii*, only slight TAL activity was detected from BoPAL2. No TAL activity was detected from BoPAL1, while BoPAL4 possessed both PAL and TAL activity [21]. The enzymes SpPAL1 and SpPAL2 were observed to catalyze the transformation of L-Tyr into *p*-coumaric acid, as illustrated in Figure 4F–I. In contrast, SpPAL3 did not exhibit the production of *p*-coumaric acid when L-Tyr was used as the substrate (Figure 4G). This difference may provide a clue to explaining why the three *SpPALs* are not close to each other on the phylogenetic tree (Figure 1). It is necessary to further investigate the functional differences among these SpPALs in *S. polyrhiza*.

Different PAL family members frequently exhibit distinct spatial and temporal expression patterns, suggesting that they may play unique yet overlapping roles in the interactions between plants and their environment [19]. In *Arabidopsis*, both *AtPAL1* and *AtPAL2* exhibit similar patterns of gene expression and have overlapping functions in the biosynthesis of flavonoid [24]. However, *AtPAL3* is expressed at very low levels during various developmental stages [37]. To date, the expression of *PAL* genes has been studied in many plants [11,38]. In our study, transcripts of *SpPALs* were found in both mature fronds and young fronds (Figure 5A,B). In the young fronds, the expression level of *SpPAL3* was the highest compared to other *SpPALs* (Figure 5B). High expression levels of *PAL* have also been found in the leaves of spruce *Picea asperata* [39], *Cuminum cyminum* L. [40], and *Salix viminalis* [41]. These results further indicate that *SpPALs* may have different functions in duckweed during various stages of development.

Meanwhile, *PALs* can be induced by many factors, such as wounding [33], UV irradiation [32], MeJA treatment [27], SA treatment [42], and so on. In the present research, duckweed plants were treated with 100 μM MeJA, and we found a minor initial increase in the expression levels of the *SpPAL1* and *SpPAL3* genes. On the tenth day, the expression levels of *SpPAL1*, *SpPAL2,* and *SpPAL3* reached their highest levels. And finally, on the twelfth day, the expression levels of these three genes were decreased (Figure 5C). These results were similar to the *PAL* expression patterns in tomato [43], *Solanum tuberosum* [44], and *Foeniculum vulgare* Mill. [45] under MeJA treatment. The possible reason for this expression pattern may be the expression of *PAL* induced by MeJA, considered as a part of the plant defense mechanisms. As the time goes on, the plant adapts to MeJA stress, leading to a decrease in the expression level of *PAL* [29,43,44]. Combined with the results of *SpPALs* expression in different tissues, *SpPAL2* may be functionally redundant, while *SpPAL3* may primarily contribute to the growth and development of duckweed.

To further confirm the function of *SpPAL3* in duckweed, we generated four *SpPAL3* overexpression transgenic lines in *L. turionifera* (Figure 6). The length of adventitious roots, frond phenotype, and fresh weight of *SpPAL3* overexpression plants were significantly different from those of WT plants (Figure 6C,D). When PAL was overexpressed in *Panax ginseng*, the phenotype of the roots in transgenic plants also changed [46]. When *IbPAL1* was overexpressed in sweet potato, it inhibited the formation of storage root [17]. These studies showed that *PAL* changed the phenotype of plant roots [17,46]. In our study, we analyzed the pigment content, soluble protein content, flavonoid content, and anthocyanin content in *SpPAL3*-overexpressed duckweeds (Figure 7). And we found that only the soluble protein content in transgenic plants has significantly changed compared to WT plants (Figure 7D). Further studies are needed to determine how *SpPAL3* regulates root structure and influence the synthesis of protein.

Currently, sequencing of several duckweed species has been completed, and the resulting data are gradually being released, which promotes a boom of research related to duckweed [47]. Successful application of gene knockout/editing technology has been reported in *Lemna* [12], which may indicate that duckweed is suitable for utilization in the field of plant synthetic biology. To develop *S. polyrhiza* into an efficient plant bioreactor for manufacturing various flavonoids using a biosynthetic biology strategy, it is necessary to clone and characterize genes involved in the biosynthetic pathway. The findings of our study provide substantial theoretical support for the functional analysis of duckweed *PAL* genes and lay the foundation for duckweed as a plant synthetic biology chassis.

## 4. Materials and Methods

### 4.1. Cultivation of Plant Materials and Application of MeJA Treatment

The sterilized *Spirodela polyrhiza* (L.) Schleid and *Lemna. turionifera* were grown under a 12 h photoperiod and cultivated in the laboratory of Nankai University (Tianjin, China). The duckweed was cultivated in a 150 mL flask with 60 mL of liquid Dakto medium at a temperature of 25 °C. The duckweed was treated with 100 μM MeJA and collected at 2, 4, 6, 8, 10, and 12 days after treatment. The sample was promptly cryogenically frozen with liquid nitrogen and then stored at a temperature of −80 °C for potential future applications.

### 4.2. Bioinformatics Analysis

The open reading frame (ORF) was identified using the ORF Finder tool available on the National Center for Biotechnology Information website. Multiple sequence alignment was performed using the DNAMAN software 6.0. A phylogenetic tree was constructed using a neighbor-joining algorithm in MEGA 7.0 software, based on *PAL* nucleotide sequences obtained from GenBank. The isoelectric point and molecular weight of SpPALs were predicted using a ProtParam online tool, which can be accessed at http://web.expasy.org/compute_pi/ (accessed on 10 July 2023). The subcellular localization prediction was conducted using an online tool, CELLO, which is accessible at http://cello.life.nctu.edu.tw (accessed on 1 September 2023). Secondary structure prediction was performed using a SOPMA online program available at https://npsa-prabi.ibcp.fr/cgi-bin/npsa_automat.pl?page=npsa_sopma.html (accessed on 12 August 2023). Homology modeling was conducted using SWISS-MODEL, which can be accessed at https://swissmodel.expasy.org (accessed on 1 September 2023). Prediction of transmembrane regions and signal peptides was conducted using a TMHMM Server v. 2.0, which can be found at http://www.cbs.dtu.dk/services/TMHMM/ (accessed on 21 August 2023), and a SignalP-5.0 Server, which is accessible at http://www.cbs.dtu.dk/services/SignalP/ (accessed on 1 September 2023), respectively.

### 4.3. Cloning of SpPAL1, SpPAL2, and SpPAL3

Sequences of *SpPAL1*, *SpPAL2*, and *SpPAL3* were retrieved from the genome database (https://phytozome.jgi.doe.gov/pz/portal.html# (accessed on 1 September 2023)). A pair of specific primers (see Supplementary Table S1) was designed using Primer Premier version 5.0 software. Total RNA extraction was performed according to the manufacturer’s instructions (Tiangen Biotech; Beijing; China). Isolated total RNA (~500 ng) was used to generate cDNA, using a reverse transcriptase kit (catalog no. RR036A; Takara Biotechnology, Kusatsu, Japan). For PCR, we used cDNA as the template with 2x Pfu MasterMix (Dye) from CWBIO in Beijing, China. Thermal cycling was conducted according to the specified protocol, which included an initial denaturation step at 95 °C for 5 min, followed by 31 cycles consisting of 30 s at 95 °C, 30 s at 58 °C, and 120 s at 72 °C, with a final extension step at 72 °C for 5 min. Subsequently, the PCR products were sequenced at the Beijing Genomics Institute (BGI) in Shenzhen, China.

### 4.4. Expression and Purification of Recombinant SpPALs in E. coli

The SpPALs were expressed in *E. coli* and purified using previously described protocols, with minor modifications [48]. The full-length ORFs of *SpPALs* cDNAs were subcloned and inserted into the *BamH*I-*Hind*III, *EcoR*I-*Hind*III, and *BamH*I-*Hind*III sites of pET-28a (Novagen, Madison, WI, USA), respectively. The plasmids generated were used to synthesize fusion proteins containing 6-His tags located at the N-terminus. These engineered plasmids, along with an empty vector used as a reference, were introduced into *E. coli* Rosetta (DE3) cells obtained from TransGen Biotech in Beijing, China. A preculture of 3 mL was incubated overnight at 37 °C in LB medium. This culture was utilized to inoculate 300 mL of fresh medium, achieving a density corresponding to an optical density (OD_600_) of 0.5 at 25 °C. The cells were then incubated for 8 h at 16 °C. Subsequently, 0.1 mM isopropyl-β-D-thiogalactopyranoside (IPTG) was administered to induce protein expression. The cells were collected through centrifugation and subsequently lysed via sonication. The recombinant proteins tagged with histidine were isolated using a His-Tag purification system from Roche in Mannheim, Germany. The protein purity was assessed through 10% SDS-PAGE analysis, while the protein concentration was quantified using the Coomassie Brilliant Blue G-250 technique. The purified proteins were used for further enzymatic tests.

### 4.5. Enzyme Activity Assays

The enzymatic activity of SpPALs was analyzed using a previously established method with slight adjustments [32]. To determine the kinetic parameters, various concentrations of L-phenylalanine (ranging from 0 to 20 mM) or L-tyrosine (ranging from 0.1 to 2.5 mM) were investigated. In order to identify the most favorable pH conditions, experiments were conducted at 37 °C for 20 min at various pH levels. In order to identify the most favorable temperature, experiments were conducted at favorable pH for a duration of 20 min, using a variety of temperatures. The experiments were conducted three times to ensure accuracy and reliability.

The purified SpPAL1-3 (4 μg) were incubated with 25 mM L-Phe or L-Tyr in 730 μL of 100 mM Tris-HCl buffer (pH 8.0) at 50 °C for 20 min, respectively. The enzyme reaction was stopped by adding 50 μL of 6 M HCl [49]. The products were further identified by HPLC. The HPLC analysis was performed using an Agilent 1200 series liquid chromatography system (Agilent, Palo Alto, CA, USA). The C18 column (4.6 mm × 150 mm) temperature was maintained at 25 °C, with a flow rate of 1.0 mL/min and an injection volume of 10 μL. The mobile phase consisted of solvent A (acetonitrile) and solvent B (0.1% formic acid in double-distilled water). Detection of chromatographic peaks for trans-cinnamic acid and *p*-coumaric acid was carried out at 290 nm and 310 nm, respectively [31].

### 4.6. Expression Analysis of SpPALs by qRT-PCR

Total RNA was extracted from all samples using the RNeasy^®^ Plant Mini Kit (Qiagen, Shanghai, China) in preparation for real-time quantitative PCR (qRT-PCR) analysis. Subsequently, cDNA synthesis was performed using the PrimeScript™ RT Master Mix (Takara, Kusatsu, Japan) following the manufacturer’s instructions. The qRT-PCR specific primers used are detailed in Supplementary Table S1. *INO1* (Spipo4G0013100) was used as an internal reference. qRT-PCR was performed on an iCycler Thermal Cycler (Bio-Rad iQ5, Hercules, CA, USA) using TB Green Premix Ex TaqII (Dalian Takara, Dalian, China) following the standard manufacturer’s protocol. The reaction mixture was heated at 95 °C for 30 s, followed by 40 cycles of polymerase chain reaction (PCR) at 95 °C for 5 s, 58 °C for 30 s, and 72 °C for 30 s. The primer pairs exhibited efficiencies ranging from 95% to 105%, and specific efficiency values were considered when determining normalized relative expression. Discrepancies in the relative expression levels of *SpPALs* were assessed using the 2^−ΔΔCT^ method.

### 4.7. Generating Transgenic SpPAL3 Duckweed Plants

The open-reading frame of *SpPAL3* was inserted into the plant gene overexpression vector to generate the expression cassette CaMV35S::*SpPAL3*::NOS. The plasmid was transformed into *Agrobacterium* EHA105 to obtain *SpPAL3* transgenic duckweed (*L. turionifera*). The method for inducing duckweed calluses, subculturing, regenerating, and transforming described by Yang et al. [50] was followed. The positive plants were confirmed by selection with 30 mg/L hygromycin, PCR, and RT-PCR. For PCR and RT-PCR, we used transgenic *SpPAL3* plants and wild-type *L. turionifera* plants cDNA as the template. The PCR and RT-PCR reagents were similar in the 4.3. RT-PCR thermal cycling was conducted according to the specified protocol, which included an initial denaturation step at 95 °C for 5 min, followed by 28 cycles consisting of 30 s at 95 °C, 30 s at 58 °C, and 120 s at 72 °C, with a final extension step at 72 °C for 5 min. The activity of PAL in transgenic *SpPAL3* plants and wild-type *L. turionifera* plants was measured using the Phenylalnine Ammonialyase-Lyase Activity Assay Kit (BC0210) from Solarbio, Beijing, China.

### 4.8. Analysis of Fresh Weight, Soluble Protein Content, Pigment Content, and Phenolic Content

For the measurements of fresh weight, pigment content, and phenolic content, transgenic *SpPAL3* and wild-type duckweed plants were cultivated in Dakto medium and harvested at different days. The method for measuring duckweed fresh weight and pigment content described by Zhu et al. [51] was followed. The soluble protein content was measured using the Protein Content Assay Kit (BC3185) from Solarbio. The method for analyzing duckweed anthocyanins and flavonoids content was based on Dupont’s protocol [52].

### 4.9. Statistical Analysis

The data are presented as the mean ± standard error of the mean, calculated from at least three biological replicates. Statistical significance was assessed through one-way ANOVA and Student’s *t*-test (* *p* < 0.05, ** *p* < 0.01).

## 5. Conclusions

In this study, the *S. polyrhiza PAL* gene family members, *SpPAL1*, *SpPAL2*, and *SpPAL3*, were successfully isolated and cloned. The full-length encoding sequence of the *SpPAL1*, *SpPAL2*, and *SpPAL3* genes were 2172 bp, 2277 bp, and 2169 bp, respectively. Multiple sequence analysis and phylogenetic relationships demonstrate that SpPALs are closely related to other monocotyledons. Furthermore, the SpPALs proteins were expressed and purified in vitro, which exhibited high PAL enzyme activity and slight TAL enzyme activity (except for *SpPAL3*). The qRT-PCR analysis showed that *SpPAL3* was expressed at the highest level in young fronds, and *SpPAL1* and *SpPAL3* were found to be sensitive to MeJA treatment. Overexpression of *SpPAL3* in *L. turionifera* could indicate its involvement in the growth and development of duckweed. The work shows the enzyme characteristics of SpPALs, which demonstrated that it is one of the alternative pathways to implement genetic alterations to control the distribution of primary and secondary metabolites in duckweed. Further investigations into the functions of SpPALs need to carried out in the future, including genetic modification and CRISPR/Cas9, which could advance our understanding of the metabolic pathways of primary and secondary metabolites in S. polyrhiza. At the same time, it is an innovative approach to enhance the levels of medicinal substances in duckweed.

## Figures and Tables

**Table 1:** Predicted physicochemical properties, subcellular localization, transmembrane helices, and signal peptide of SpPALs.

Gene	Gene Length (bp)	Protein Length (aa)	Molecular Weight (kDa)	pI	Subcellular Location	Transmembrane Helices	Signal Peptide
*SpPAL1*	2172	724	78.03	6.12	Cytoplasmic (2.091)	No	No
*SpPAL* *2*	2277	759	83.59	6.01	Cytoplasmic (1.892)	No	No
*SpPAL* *3*	2169	723	79.02	6.35	Cytoplasmic (1.751)	No	No

**Table 2:** Biochemical characterization of SpPALs.

Substrate	Enzyme	Km (μM)	Kcat (s^−1^)	Kcat/Km (s^−1^ M^−1^)	pH Optima	Temperature Optima (°C)
L-Phe	SpPAL1	252	9.83	39,008	8	50
	SpPAL2	345	7	20,290	8	60
	SpPAL3	632	3.35	5301	7.5	50
L-Tyr	SpPAL1	1294	0.136	105	NO	NO
	SpPAL2	8718	0.313	36	NO	NO
	SpPAL3	ND	ND	ND	NO	NO

ND: That means not detected; NO: That means not conducted.

## Data Availability

All data supporting this study are included in the article.

## Supplementary Materials

The following supporting information can be downloaded at: https://www.mdpi.com/article/10.3390/plants13182607/s1, Figure S1: Multiple alignment analyses of *SpPALs* in duckweeds; Figure S2: Predicted tertiary structure of SpPAL1, SpPAL2 and SpPAL3 proteins; Table S1: Primer sequence used in this research; Table S2: All 20 MEME motif sequences in SpPAL proteins.
