# Supplementary material for: Enzymatic Characterization of SpPAL Genes in S. polyrhiza and Overexpression of the SpPAL3
Source: Plants (Basel). 2024 Sep 18;13(18):2607. doi: 10.3390/plants13182607 (PMC11435183; doi:10.3390/plants13182607)
Supplement: Supplementary file 1 [file plants-13-02607-s001.zip › plants-3143609-supplementary.pdf]

Supplementary File

Supplementary Table S1

Table S1. Primer sequence used in this research

Primer name	Primer usage	Primer sequences (5'-3')
SpPAL1-F	ORF cloning	**CGCGGATCC**ATGGAGGCCGCCGGCCA
SpPAL1-R		**CCCAAGCTT**CTAGCAGATGGGGACGGG
SpPAL2-F		**CCGGAATTC**ATGGCGGCGCAGACGAAC
SpPAL2-R		**CCCAAGCTT**CTAGTTGTTCCAGTACCCCCTGCCA
SpPAL3-F		**CGCGGATCC**ATGGAAGCCGCCACCCAAGTT
SpPAL3-R		**CCCAAGCTT**CTAGCAGATGGGTACGGGGGACC
SpPAL1-qRTF	qRT-PCR	CAGGTCCTACCCGCTCTACAA
SpPAL1-qRTR		CAATCCCGCAAGCACTCG
SpPAL2-qRTF		CATCCGCTGGGAGGTTCT
SpPAL2-qRTR		TCCTTCGCCTGGAGTTCG
SpPAL3-qRTF		ATCGGCAAGCTCATGTTCG
SpPAL3-qRTR		CCGTAGTCCAGGCTAGGGTT
INO1-qRTF		ACTCCTTCCACCCAGTAGCC
INO1-qRTR		TCTCCAGCATCGCCCTCT

Bold case letters indicate the protective bases and restriction enzyme sites.

Supplementary Table S2

Table S2. All 20 MEME motif sequences in SpPAL proteins.

Motif	Width(aa)	Best possible match
motif 1	50	NPSLDYGFKGAEIAMAAYCSELQFLANPVTNHVQSAEQHNQDVNSLGLIS
motif 2	50	FCEVMQGKPEFTDHLTHKLKHHPGQIEAAAIMEHILEGSSYMKMAKKIHE
motif 3	50	PLQKPKQDRYALRTSPQWLGPQVEVLRQATKSIEREINSVNDNPLIDVSR
motif 4	50	KALHGGNFQGTPIGVSMDNSRLAIAAIGKLMFAQISELVNDFYNNGLPSN
motif 5	50	SSDWVMDSMNRGTDSYGVTTGFGATSHRRTKQGAALQKELIRFLNAGIFG

motif 6	50	RINTLLQGYSGIRFEIMEAITALLNHHVTPCLPLRGTITASGDLVPLSYI
motif 7	50	CRSYPLYKFVREELGTAMLTGEKVRSPGEEFDKVFTAISQGKVIDPLFNC
motif 8	50	RKTAEAVEILKLMTSTYLVALCQAVDLRHLEENLKNAVKNTVSQVAKRVL
motif 9	48	NGELHPSRFCEKDLLKVVDREYVFAYADDPCLSTYPLMQKLRQVLVEH
motif 10	41	FFELQPKEGLALVNGTAVGSGLASMVLFEANILAVLAEVLS
motif 11	30	DPLNWGAAAESLSGSHLEEVKRMVEEFRQP
motif 12	46	KEKDSATSIFQKVTAFEEELKMALPKEVEAAREAVEKGAPAIPNRI
motif 13	9	WNGAPVPIC
motif 14	29	MLTGRPNARAVNAAGEKVSAQEAFQQAGI
motif 15	15	NTTLPQSATRAAMLV
motif 17	7	EGFCLKP
motif 18	16	VQLEGADLKISQVAAV
motif 19	14	LRVELADSARPRVK
motif 20	6	MEAAGQ

Supplementary Figure S1

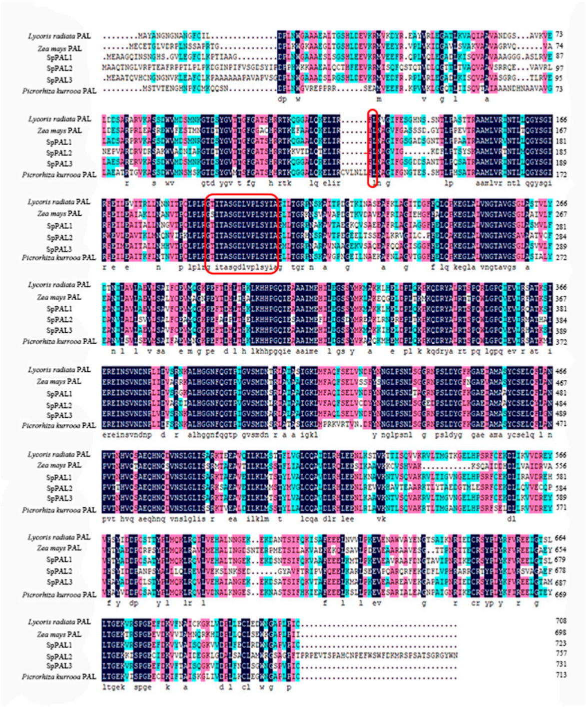

Figure S1. Multiple alignment analyses of SpPALs in duckweeds.

Multiple alignment of the SpPAL1, SpPAL2, and SpPAL3 proteins with other plant PAL proteins. The amino acid sequences are from *Lycoris radiata* (AWW24969.1, PAL), *Zea mays* (ONM07141.1, PAL1), and *Picrorhiza kurrooa* (AGA84059.1, PAL). The preserved patterns of the *PALs* are denoted by red boxes. The strongly preserved elements are emphasized in black, whereas comparable elements are highlighted in shades of pink and blue.

Supplementary Figure S2

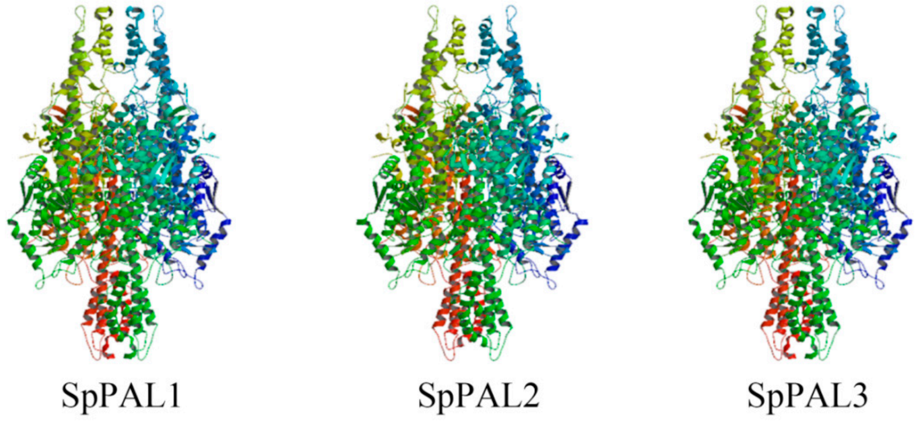

Figure S2. Predicted tertiary structure of SpPAL1， SpPAL1 and SpPAL3 proteins.
